# DNA Methylation Analysis of Allotetraploid Hybrids of Red Crucian Carp (*Carassius auratus* red var.) and Common Carp (*Cyprinus carpio* L.)

**DOI:** 10.1371/journal.pone.0056409

**Published:** 2013-02-15

**Authors:** Jun Xiao, Can Song, Shaojun Liu, Min Tao, Jie Hu, Jun Wang, Wei Liu, Ming Zeng, Yun Liu

**Affiliations:** Key Laboratory of Protein Chemistry and Fish Developmental Biology of the Ministry of Education of China, College of Life Sciences, Hunan Normal University, Changsha, China; The University of Arizona, United States of America

## Abstract

Hybridization and polyploidization may lead to divergence in adaptation and boost speciation in angiosperms and some lower animals. Epigenetic change plays a significant role in the formation and adaptation of polyploidy. Studies of the effects of methylation on genomic recombination and gene expression in allopolyploid plants have achieved good progress. However, relevant advances in polyploid animals have been relatively slower. In the present study, we used the bisexual, fertile, genetically stable allotetraploid generated by hybridization of *Carassius auratus* red var. and *Cyprinus carpio* L. to investigate cytosine methylation level using methylation-sensitive amplification polymorphism (MSAP) analysis. We observed 38.31% of the methylation changes in the allotetraploid compared with the parents at 355 randomly selected CCGG sites. In terms of methylation status, these results indicate that the level of methylation modification in the allotetraploid may have increased relative to that in the parents. We also found that the major methylation changes were hypermethylation on some genomic fragments and genes related to metabolism or cell cycle regulation. These results provide circumstantial evidence that DNA methylation might be related to the gene expression and phenotype variation in allotetraploid hybrids. Our study partly fulfils the need for epigenetic research in polyploid animals, and provides evidence for the epigenetic regulation of allopolyploids.

## Introduction

Since Ohno [1] put forward the hypothesis of genome duplication in 1970, researchers have paid much attention to polyploidy in animals. Now the 2R hypothesis, two rounds of polyploidy occurring during the early evolution of the vertebrate lineage, is widely recognized. Polyploids are very common among fish and amphibians [2]. In particular, the ray-finned fishes experienced another genome duplication, the fish-specific genome duplication (FSGD or 3R) [3], [4]. While polyploidization is a widespread mechanism for speciation and adaptation, hybridization is also a catalyst for evolutionary innovations [5]. Distant hybridizations and the accompanying combination of different genomes speed up the formation of allopolyploids, which demonstrate a selective advantage over their diploid progenitors [6].

Genome merging and doubling brings about plenty of gene redundancy, so polyploids must go through genomic recombination and gene expression changes to pass through a bottleneck of instability [7], [8]. Although these genetic events contribute to genome evolution of newly formed polyploids, an increasing number of researchers are focusing on the epigenetic mechanisms that underlie the expression changes in allopolyploids [9]. As a common epigenetic phenomenon, alterations in DNA methylation could regulate gene expression, or other important epigenetic processes including dosage compensation, genomic imprinting, nucleolar dominance, de-repression of dormant transposable elements, and alterations in chromatin structure among others[6], [10]–[12]. These changes corroborate the “genomic shock” hypothesis and are supported by experimental evidence in several allopolyploid plants, such as *Arabidopsis*, wheat, *Senecio*, *Oryza sativa*, and orchid [13]–[16]. However, because allopolyploid animals are relatively rare, especially among relevant model organisms, the epigenetic influence in polyploid animals is poorly understood.

In our previous study, We found that the distant hybridization of red crucian carp (RCC for short, *Carassius auratus* red var., 2*n* = 100) (♀) and common carp (CC for short, *Cyprinus carpio* L., 2*n* = 100) (♂), the offspring F_1_ and F_2_ were diploid hybrids [17]. Interestingly, the F_2_ hybrids were able to generate unreduced diploid gametes. Then, self-mating of the F_2_ hybrids produced bisexual fertile allotetraploid hybrids (abbreviated as 4*n*AT) in the F_3_ generation. Successive self-mating has continued to F_22_, and F_3_–F_22_ hybrids form a bisexual fertile, genetically stable allotetraploid population [18]. This tends to form a new species and provides an excellent polyploidy model system for genetic and epigenetic studies in animals. In recent years, several investigations on the genetic relationship between allotetraploids and their parents, at the cellular and molecular level, have been performed, which partly explained the gene expression and phenotype change that happened following hybridization and polyploidization in allotetraploid hybrids [17]. However, these studies do not illustrate the reason that these changes occured in the allotetraploid strain. In this study, we focused on the genetic change in DNA methylation in these allotetraploids, because it is the predominant mechanism underlying epigenetic regulation.

DNA methylation occurs in symmetrical CpG or CpNpG contexts as well as in non-symmetrical contexts, and is mediated by DNA methyltransferases, which catalyse the transfer of a methyl group from S-adenosyl-methionine (AdoMet) to cytosine pyrimidine ring carbon 5 within the DNA helix [19], [20]. The methylation-sensitive amplification polymorphism (MSAP) technique is a modification of the amplified fragment length polymorphism (AFLP) technique [21], and represents a simple, efficient and reliable technique for the detection of cytosine methylation alterations in terms of both level and pattern, from a genome-wide perspective [22]. This method uses a pair of isoschizomers, MspI and HpaII, which recognize the same restriction site (CCGG) but have different sensitivities to certain cytosine methylation statuses. MspI is active if the internal cytosine is fully (double-strand) methylated, whereas HpaII will cut if the external cytosine is hemi (single-strand) methylated [23]. Thus, for a given DNA sample, full methylation of the internal cytosine or hemi-methylation of the external cytosine at the assayed CCGG sites can be unequivocally distinguished [24]. However, it should be noted that, because MspI and HpaII cannot differentiate among several other methylation states, including double-strand methylated mCmCGG, double-strand methylated mCCGG and single-strand methylated CmCGG, the methylation percentages at the CCGG sites calculated by MSAP should be lower than the total absolute values [24]. Even so, MSAP analysis is still applicable.

Here, we investigated the genome-wide cytosine methylation pattern in F_18_ individuals of the 4*n*AT strain, and the original parents, using MSAP analysis. The results imply that the methylation level in allotetraploids may have been increased relative to that in the parents. In addition, our findings may help us recognize DNA methylation modifications of some genes and sequences, and could contribute to an understanding of the effects of epigenetic change on allotetraploid formation.

## Results

### MSAP Analysis

In this work, we made use of MSAP analysis to characterize the cytosine methylation pattern in 4*n*AT hybrids and their parents, red crucian carp and common carp, and to analyze the differences in methylation patterns among them. After a series of procedures, including double-restriction with EcoRI/MspI or EcoRI/HpaII, adapter ligation, and then amplification, methylated bands would be reflected in gels with four different kinds of patterns. Band amplification after both MspI and HpaII treatments indicated the occurrence of an unmethylated CCGG site. Band amplification after MspI treatment only indicated the occurrence of an internally fully-methylated CCGG site (CmCGG). Band amplification after HpaII treatment only indicated the occurrence of an externally hemi-methylated CCGG site (mCCGG). An absence of bands after both treatments indicated full methylation of all cytosine, full methylation of the external cytosine, hemi-methylation of the internal cytosine, or nonexistence of the site (caused by a genetic mutation).

**Figure 1:**
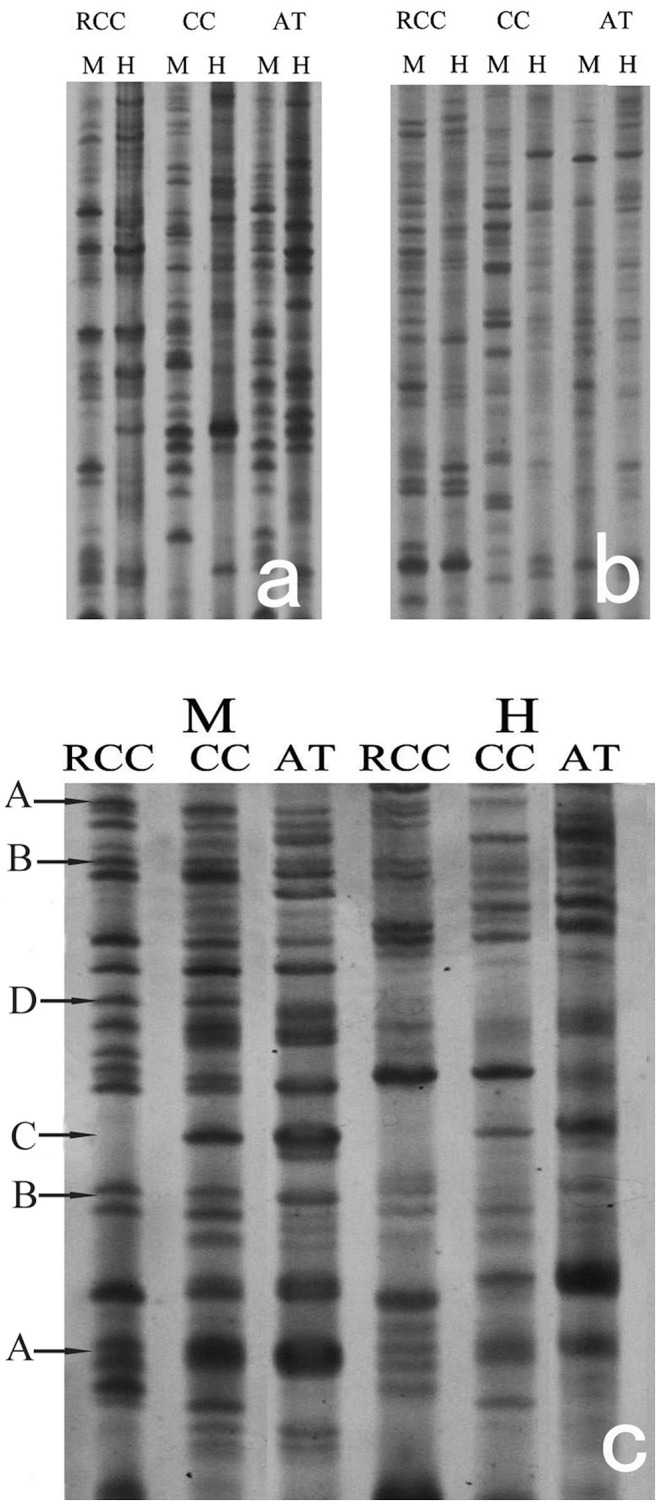
Examples of MSAP analysis. (a) Primer combination AGC/TCTG; (b) Primer combination AGC/TTG; (c) Primer combination AAC/TGC. A, B, C and D represent bands corresponding to the methylation types with these labels in Table S1.

Among the five EcoRI and seven MspI–HpaII primer pairs we tested, we selected six primer combinations for further study, based on their amplification of favorable polymorphisms and clear banding patterns. In total, 355 groups of bands were amplified from these six primer combinations for RCC, CC and AT hybrids. The same results were obtained in three experiments, and there was a high degree of homogeneity in the methylation state from a single specimen (Figure 1).

### Classification of the Cytosine Methylation Pattern

The 355 groups of bands were organized into 61 categories. To simplify these mass data, we divided them into seven major classes (A–G) in accordance with the inheritance model or the variation level between the parent(s) and the hybrid. As shown in Table S1, 12.96% of methylation patterns were transmitted to the allotetraploid from both parents in class A. In classes B and C, respectively, 23.94% of the sites showed maternal methylation patterns and 24.79% showed paternal methylation patterns in the hybrid. The fact that 61.69% of the methylation patterns fell into classes A, B and C illustrates that the allotetraploid hybrid inherits most of these patterns from the parent(s) in a Mendelian way. The remaining patterns (38.31%) represent the variant types with methylation patterns between those of the parents and the allotetraploids. That is to say, the methylation patterns in classes D through G were locus-specific in the allotetraploids compared with the parents. Eleven types representing 13.8% of the sites fell into class D, showing hypermethylation (an increase in methylation levels) in the allotetraploids. Some loci in the allotetraploids showed a decrease in methylation levels (hypomethylation), and the 9.58% showing this pattern were subgrouped into class E. In the four types (7.89%) in class F, the methylation level in the allotetraploids was between that in one parent and that in the other. Finally, some variant types did not belong to any of the classes from A to F; these were classified into class G, with a 7.04% overlap of CCGG sites.

### Comparison of the Cytosine Methylation Status

The numbers of loci with each possible methylation status are shown in Table 1. In term of the ratio between RCC and CC, all the four kinds of methylation status were not significantly different (P>0.05) from the 1∶1 ratio, revealing that the RCC and the CC possess the same degree of methylation at the 355 randomly selected CCGG sites. In terms of the ratios between AT and RCC, the ratio for the number of methylations at internal cytosine was significantly different from the 1∶1 ratio (P<0.05). In term of the ratios between AT and CC, the ratio for the number of methylations at an internal cytosine was also significantly different from the 1∶1 ratio (P<0.05). Both ratios (1.35 and 1.46 for AT/RCC and AT/CC, respectively) for the number of methylations at internal cytosine were significantly higher than 1, indicating that the level of methylation at internal cytosine is significantly increased in AT hybrids compared with the original parents (P<0.05). All the probabilities were calculated by using X^2^ test with Yate’s correction.

**Table 1:** Methylation pattern and corresponding status.

PatternMspI HpaII	Methylation status	Material			Ratio			
		RCC	CC	AT	Observed			Expected
					RCC/CC	AT/RCC	AT/CC	
1 1	u	140	134	114	1.04^a^	0.81^a^	0.85^a^	1
1 0	i	80	74	108	1.08^a^	1.35^b^	1.46^b^	1
0 1	e	49	57	56	0.86^a^	1.14^a^	0.98^a^	1
0 0	f	86	90	77	0.96^a^	0.90^a^	0.86^a^	1
Total		355	355	355				

u = unmethylated, i = methylated at internal cytosine (C5mCGG), e = methylated at external cytosine (5mCCGG), f = fully methylated (5mC5mCGG) at all cytosines, or fully methylated at external cytosines, hemi-methylated internal cytosines or an absence of CCGG sites.

a The observed ratio was not significantly different (P>0.05) from the expected ratio,

b The observed ratio was significantly different (P<0.05) from the expected ratio.

### Analysis of Polymorphic Fragment Sequences

Nineteen fragments that displayed variable cytosine methylation patterns among allotetraploids and parents were eluted from the gel, reamplified, and sequenced. Among them, sixteen satisfactory sequences were obtained and contrasted against the GenBank database using the NCBI BLAST tools. Of the 16 fragments tested, eleven showed similarity to characterized genome regions of zebrafish and other fishes (Table 2). These sequences include the genes encoding ATPase polypeptide, Rho guanine nucleotide exchange factor, metabotropic glutamate receptor 4, and some supposed novel proteins, as well as the microsatellite J42 sequence. Because the sequence of the zebrafish genome has not yet been completely solved, there was insufficient information available for us to locate our sequences. Thus, we could locate only one polymorphic sequence within an intron, one within an exon, and two crossing intron–exon boundaries.

**Table 2:** Sequences of methylated fragments and database search results.

MSAPFragment	Primer^a^(E/M-H)	Length^b^(bp)	GenBankAccession No.	SequenceHomology	Identity^c^	BlastE score
1-1-H-1	AC/TGC	377	AL590134.7	a novel protein(Zebrafish)	81%	2e-52
1-1-M-1	AC/TGC	614	BX294126.10	ATPase, Na+/K+ transporting, alpha 2a Polypeptide(Zebrafish)	78%	1e-11
1-1-M-2	AC/TGC	443	BX511005.6	a novel protein similar to vertebrate peroxisomal membraneprotein 2(Zebrafish)	81%	8e-57
1-1-M-5	AC/TGC	190	XM_690948.4	Rho guanine nucleotide exchange factor(GEF)10-like(LOC567649),mRNA(Zebrafish)	90%	6e-08
1-1-M-6	AC/TGC	170	BX537350.25	a novel protein (Zebrafish)	74%	1e-09
2-1-M-1	CG/TGC	487	FQ310506.1	Metabotropic glutamate receptor 4 (European seabass)	79%	4e-60
5-3-M-2	GC/TCAC	549	AY115100.1	microsatellite J42 sequence(Silver crucian carp)	76%	1e-29
5-3-M-3	GC/TCAC	410	BX784023.10	cryptochrome 2(Zebrafish)	72%	3e-16
5-3-M-4	GC/TCAC	301	AL953858.10	a novel protein(Zebrafish)	75%	3e-21
5-3-M-5	GC/TCAC	278	AB040746.1	c-MYC(Goldfish)	94%	3e-08
5-3-M-6	GC/TCAC	246	AL928951.17	A novel protein similar to vertebrate cyclin M2(Zebrafish)	77%	2e-35

a Nucleotide extensions of primers used in MSAP amplification, listed with the EcoRI primers first and the MspI or HpaII primers second.

b Length of sequence.

c Nucleotide identity to the BLASTN sequence.

## Discussion

Our previous research has shown that allotetraploids derived from red crucian carp (♀) and common carp (♂) maintain a stable inheritance of tetraploidy from generation to generation after formation, and that they have undergone various changes to their genotypes and phenotypes compared with the parents [17], [25]. Individual allotetraploids have the same karyotype formula and produce diploid gametes. Phylogenetic analysis of *Sox9a* indicated that allotetraploids show unique traits as well as heterozygosity [26], [27]. Random amplified polymorphic DNA analysis has demonstrated the genetic stability of the allotetraploid population. Based on inter-simple sequence repeat and AFLP markers, and cloning of cyclin genes, Liu L G *et al.* [28] also showed that the allotetraploids stably maintain genetic characteristics from their parents after multiple generations. In addition, the non-additive genetic changes found in the allotetraploids could be an adjustment to the genomic shock from heterozygosity and polyploidy, allowing for the maintenance of genetic stability. Although many molecular genetic studies have been carried out, the reasons for the adaption and evolutionary success of the 4*n*AT hybrids are not yet clear. In this study, epigenetic modifications in genomic DNA of allotetraploid hybrids were studied. By comparing the methylation patterns in allotetraploid offspring and their original parents, the role played by the effects of epigenetic change in their adaption and evolutionary success can be brought to light.

The methylation patterns in allotetraploids were diverse. Among the 355 randomly selected CCGG, sites analyzed, most (61.69%) of the cytosine methylation patterns appeared to follow simple Mendelian inheritance. This suggests that the allotetraploids inherited some of the methylation characteristics from their parent(s) and stably maintained the pattern from one generation to another, just like the genetic characteristics shown by AFLP analysis [28]. Cytosine methylation alterations including hyper- and demethylation as well as conversions (classes D–G) also occurred in allotetraploids, accounting for the remaining (38.31%) cytosine methylation patterns. Previous studies have indicated that hybridization and chromosome doubling have led to genetic changes in allotetraploid offspring [17], [28]. Data from 454 sequencing experiments targeting the transcriptome suggested the existence of rapid mutation and positive selection acting in at least 41 genes under strong natural selection pressure (unpublished data). Recent findings have suggested that the accelerated evolution and enhanced fitness of allopolyploids may also be the result of an epigenetic influence, and that genomic cytosine methylation changes are a major manifestation of this [15]. An analysis of different allotetraploid sibling orchid taxa demonstrated that ecological divergence and adaptation were largely due to epigenetic effects, which modulate gene expression under an environmental influence [16]. DNA methylation pattern changes have some epigenetic effects. A series of studies in polyploid plants, such as *Brassica* [29], wheat [14], *Arabidopsis* [13], [30], [31], triticale and rice [32] have demonstrated this. The results of these studies have suggested that DNA methylation could activate mobile elements and silence redundant genes, leading to epigenetic phenomena including nucleolar dominance and dosage compensation [33], [34]. In our work, there were significantly (P<0.05) more methylated CpG loci in the allotetraploid than there were in RCC and CC parents at the 355 CCGG sites, observed, which implies that the methylation level in allotetraploids has increased relative to that in the parents. This may be essential to ensure normal growth and development of the 4*n*AT hybrids. We propose that the percentage of methylation changes in 4*n*AT hybrids (38.31%) is perhaps related to the new phenotypes in the hybrid offspring. We have reason to believe that, as a major epigenetic code, the systematicity and complication of methylation mode in 4*n*AT is important for controlling the development of allotetraploids, making the population stable and benefiting their evolution.

The 355 CCGG sites analyzed in this manuscript were selected from the products of six selective primer pair combinations (with 1 to 3 random selective nucleotides). The random selective nucleotides can reduce the number of PCR products and thereby simplify the MSAP map. Although the approach we used cannot be used to investigate the methylation status at all CCGG sites in the genome, it can give some insight into the methylation status of the samples via analysis of random sites. As shown in Table 1, RCC and CC display the same methylation trends at the 355 randomly selected CCGG sites (P>0.05), indicating that the methylation status at these sites might be representative of the methylation status in different individuals. Therefore, we believe that the 355 sites analyzed in this study are sufficient enough to predict the overall trend of methylation status changes between AT hybrids and the parents at the genome level.

One important reason for the programmed global epigenetic control of genome and gene expression is variable DNA methylation and its downstream effects. Several studies [6] have demonstrated that hypermethylation is characteristic of euchromatic gene silencing. Activation of transposable elements (TEs) is accompanied by genome-wide demethylation, suggesting epigenetic repression of the element in parental species and de-repression in the hybrids. In our experiment, 13.80% of the CpG loci were detected to be hypermethylated, and 9.58% were hypomethylated. The sequences involved in variable methylation status were sequenced and found to be homologous to some metabolism- or cell cycle regulation-related genes, including ATPase, peroxisomal membrane protein 2, metabotropic glutamate receptor 4 and cyclin M2, and some repetitive sequences, such as the microsatellite J42 sequence. The sequences involved in metabolism, disease resistance, cell cycle regulation and repetitive elements were reported to be concerned with silenced or lost genes in allopolyploid wheat or *Arabidopsis* [13], [14], [35]. In addition, the activated genes were all retroelements, but TEs have not yet been found in our work. The different methylation levels of the related genes in allopolyploids and their parents may exert an influence on gene expression. Take *Arabidopsis thaliana* as an example. Allotetraploid offspring display flowering time, fertility and morphology variations, and epiallelic variations could contribute to heritable changes in complex phenotypes [15], [35], [36]; in addition, artificial demethylation further exaggerates morphology abnormalities [13]. The phenotypes of the 4*n*AT hybrids display heterozygosity and some varied traits. In 4*n*AT, the numbers of vertebrae, numbers of lateral line scales, lengths of beard, sizes of cells and growth rates are distinct from those of the parents [17]. Thus, we speculate that the variation in 4*n*AT hybrids may result from changes in gene expression and phenotypes owing to changes in DNA methylation. Further evidence is needed to show this conclusively, and other factors may also play roles, with genetic and epigenetic mechanisms acting together.

The nonadditive methylation changes (38.31%) in 4*n*AT are obviously greater than those in allotetraploid plants (for example, 8.3% in *Arabidopsis* [13] and 9% in *Brassica*) [37]. Hegarty *et al.* [15] suggested the ploidy and genome size were positively related to the level of methylation changes. The 4*n*AT genome is much larger than that of plants with the same ploidy, and the genetic material content in 4*n*AT is greater than that even in plants with higher ploidy. It is possible that other factors could take part. Compared with plants, the genetic regulation in animals is more sophisticated. Based on the above findings, we speculate that the higher degree of methylation change in polyploid animals may be common.

When discussing the above phenomenon, a question concerning the possible reasons for the obvious methylation changes in allopolyploids needs to be answered. In other words, it is necessary to determine whether the changes are triggered by hybridization or by genome duplication, or both. In interspecific *Xenopus* F_1_ hybrids [38], 20.9% of methylated fragments were hybrid-unique; these alterations originated solely from hybridization. Similar results showing that hybridization rather than polyploidy leads to most of the methylation changes have been reported in *Spartina anglica* [39] and *Arabidopsis thaliana* [13], while the degree of divergence from the parents is supposed to play a role. In *Senecio* [15], nonadditive methylation was a consequence of a genome merger in the triploid hybrid *S. x baxteri*, but the polyploidization resulted in a shift from non-additivity to additivity in allohexaploid derivatives. However, evidence in triploid dandelion lineages indicated that ploidy change alone can trigger methylation variation [40]. In this study, because the allotetraploid material was from an F_18_ individual, we cannot answer this question. Regardless, in allopolyploids, genome merger and doubling can both provide epigenetic novelties for adaptive evolution to some degree.

### Conclusions

Despite growing evidence that hybridization and accompanying polyploidization is an important driver of speciation and evolution in lower vertebrates, the mystery of the harmonious coexistence of multiple incongruous genomes within one nucleus has not yet been revealed. The epigenetic effects caused by DNA methylation changes are of great importance for genome remodeling and regulation of gene expression. Our work is the first to report the effects of DNA methylation in polyploid fish hybrids, making it the first such study in vertebrates. The mechanisms of DNA methylation undoubtedly play a role in the formation and adaptation of allopolyploid fish, and further benefit the evolution of this population. Certainly, further work is required. We plan to use the F_1_ hybrid of the red crucian carp and common carp, and the diploid gynogenetic clonal line [17] to carry out a deeper MSAP analysis, with the aim of explaining the reason (hybridization or genome duplication) for the methylation changes. High throughput sequencing and other high-resolution approaches are needed to provide direct evidence for the regulation of related sequences by modification of methylation.

## Methods

### Sample Collection and DNA Extraction

The Administration of Affairs Concerning Animal Experimentation of China guidelines state that approval from the Science and Technology Bureau of China and the Department of Wildlife Administration of China is not necessary when the fish in question are not rare or near extinction (first-class or second-class state protection level). Therefore, approval was not required for the experiments conducted in this paper.

Individual allotetraploid hybrids, red crucian carp (♀) and common carp (♂) were randomly selected. Total genomic DNA was isolated from peripheral blood cells according to standard phenol chloroform extraction procedures [41]. The experimental fishes were obtained from the Engineering Center of Polyploid Fish Breeding of the Education Ministry, Hunan Normal University.

### MSAP

We followed a modified AFLP protocol developed to assay DNA methylation. This adaptation incorporates the use of methylation-sensitive isoschizomers, MspI and HpaII, which were employed as frequent-cut restriction enzymes, and EcoRI was used as a rare-cut restriction enzyme.

The adapters for EcoRI were the same as those used in the AFLP protocol [21]. The adapters for MspI–HpaII digest fragments were designed according to Xu *et al*. [42]. The adapter sequences are shown in Table 3.

**Table 3:** Adapter sequences in the ligation reaction.

Adaptor	Sequences
EcoRI	5-CTCGTAGACTGCGTACC-33-CATCTGACGCATGGTTAA-5
MspI–HpaII	5-GACGATGAGTCTAGAA-33- CTACTCAGATCTTGC-5

All of the primers designed for the EcoRI fragments had the same core and enzyme-specific sequence (5′-GACTGCGTACCAATTC-3′). The EcoRI primers used in the pre-amplification reaction had only one selective nucleotide (E-A), while the primers for the selective amplification had three selective nucleotides (E-AAC, AAG, ACG, ACT, AGC). The core and enzyme-specific sequence in the primers complementary to the HpaII-MspI adapters was 5′-GATGAGTCTAGAACGG-3′. The primers used in the pre-amplification reaction had one selective nucleotide (M–H+T) and those used in the selective amplification had three or four selective ones (M–H +TGC, TTC, TCAC, TAC, TAG, TCTG, TTG). Adaptors and primers were synthesized by Sangon Inc. (Shanghai, China).

The quality of digested DNA can influence the success of methylation analysis [43]. To obtain completely digested genomic DNA products, aliquots of 2 µl (≤1 µg) of genomic DNA were incubated for 2 h at 37°C in a solution containing 2 µl of 10× NEBuffer4, 40 U of EcoRI and 40 U of HpaII, or 2 µl of 10× NEBuffer1, 40 U of EcoRI and 40 U of MspI in a final volume of 20 µl. Two microliters of this reaction mixture was loaded onto a 1.5% agarose gel, electrophoresed and stained with ethidium bromide to estimate the extent of digestion. Then, fragments were ligated with adapters complementary to the EcoRI or MspI/HpaII cohesive ends by adding the following mix to the 20 µl total volume: 10 µl of digestion reaction system, 2 µl of 10× Buffer, 2 Units of T4 DNA ligase, 3 pmol of EcoRI adapters and 30 pmol of MspI–HpaII adapters. The reaction was incubated overnight at 16°C, and then denatured for 8 min at 65°C. All of the restriction enzymes, namely, EcoRI, MspI, and HpaII were purchased from New England Biolabs Inc. (Beverly, MA). T4 DNA ligase came from Fermentas. The pre-amplification was performed using 1 µl of the ligation products, 0.6 µl of EcoRI primer, and 0.6 µl of MspI–HpaII primer, in a final volume of 20 µl containing 2 µl of PCR buffer, 1.2 µl of MgCl_2_ (25 mM), 1.6 µl dNTP (2.5 mM), and 1 U of Taq polymerase. The PCR reactions were performed using the following program: 5 min at 94°C, and 30 cycles consisting of 30 sec at 94°C, 1 min at 56°C, and 1 min at 72°C, and a final extension step of 7 min at 72°C. The pre-amplification products were diluted 1∶50 (v/v) and 0.5 µl was used in the selective amplification reaction with the EcoRI and MspI–HpaII primers in a final volume of 20 µl. The other components were the same as in the pre-amplification reactions. The selective amplification consisted of an initial denaturation at 94°C for 2 min, 12 cycles of 94°C for 30 sec, 65°C for 30 sec (reduced each cycle by 0.7°C), and 72°C for 1 min, followed by 23 cycles at 94°C for 30 sec, 56°C for 30 sec, and 72°C for 1 min. The final cycle was followed by an extension step at 72°C for 7 min. PCR products were resolved on 6% denaturing polyacrylamide gels. Gels were silver-stained, dried and scanned for data registration.

### Statistical Analysis

On the basis of the different patterns presented in the MspI and HpaII lanes, the possible methylation statuses were classified into four kinds. There were: unmethylated (u), methylated at an internal cytosine (i), methylated at an external cytosine (e) and fully methylated at all cytosines, or fully methylated at external cytosines, hemi-methylated internal cytosines or an absence of CCGG sites (f). To calculate the probabilities of the ratios for each kind of methylation status between AT hybrids and RCC, and between AT hybrids and CC, as well as those between RCC and CC, the X^2^ test with Yate’s correction was used to test deviations from expected ratio.

### DNA Fragment Isolation and Cloning

MSAP fragments were carefully excised from gels using a sterile scalpel, eluted by rehydrating the gel in boiling water for 5 min and reamplified using the same primers under the conditions used for selective amplification. The sizes of the PCR products were verified by agarose gel electrophoresis and the products were then purified using a Gel Extraction Kit (Sangon, Shanghai, China), cloned into the pMD18-T vector (TaKaRa), and transferred into *E. coli* DH5α. The DNA fragments inserted into the pMD18-T vector were sequenced using an automated DNA sequencer (ABI PRISM 3730). Searches for genomic sequences showing similarity to the sequences obtained were performed using the Advanced BLAST program at the National Center for Biotechnology Information website.

## Supporting Information

Table S1 Methylation type and frequency at CCGG sites in RCC, CC and AT. The value 1 represents the presence of a band, while 0 represents the absence of a band. So, an amplification pattern of type 11 corresponds to samples displaying bands in both the MspI and HpaII lanes. Amplification patterns of the type 10 correspond to samples showing an amplified band after restriction with MspI but not after restriction with HpaII. Pattern 01 corresponds to samples displaying an amplified band after restriction with HpaII but not after restriction with MspI. Pattern 00 indicates no band amplified after restriction with either isoschizomer.
(DOC)
