# Supplementary material for: DNA Methylation Analysis of Allotetraploid Hybrids of Red Crucian Carp (Carassius auratus red var.) and Common Carp (Cyprinus carpio L.)
Source: PLoS One. 2013 Feb 15;8(2):e56409. doi: 10.1371/journal.pone.0056409 (PMC3574156; doi:10.1371/journal.pone.0056409)
Supplement: Table S1 — Methylation type and frequency at CCGG sites in RCC, CC and AT. The value 1 represents the presence of a band, while 0 represents the absence of a band. So, an amplification pattern of type 11 corresponds to samples displaying bands in both the MspI and HpaII lanes. Amplification patterns of the type 10 correspond to samples showing an amplified band after restriction with MspI but not after restriction with HpaII. Pattern 01 corresponds to samples displaying an amplified band after restriction with HpaII but not after restriction with MspI. Pattern 00 indicates no band amplified after restriction with either isoschizomer. (DOC) [file pone.0056409.s001.doc]

Table S1 Methylation type and frequency at CCGG sites in RCC, CC and AT.

| Type | MspI HpaII | | | Bands | Percentage |
| --- | --- | --- | --- | --- | --- |
|  | RCC CC AT | | |  |  |
| A | 11 | 11 | 11 | 32 |  |
|  | 01 | 01 | 01 | 5 |  |
|  | 10 | 10 | 10 | 9 |  |
| Subtotal |  |  |  | 46 | 12.96% |
| B | 10 | 11 | 10 | 11 |  |
|  | 11 | 00 | 11 | 10 |  |
|  | 00 | 10 | 00 | 12 |  |
|  | 00 | 11 | 00 | 11 |  |
|  | 11 | 01 | 11 | 11 |  |
|  | 10 | 00 | 10 | 10 |  |
|  | 01 | 00 | 01 | 6 |  |
|  | 11 | 10 | 11 | 8 |  |
|  | 01 | 10 | 01 | 1 |  |
|  | 01 | 11 | 01 | 1 |  |
|  | 00 | 01 | 00 | 1 |  |
|  | 10 | 01 | 10 | 3 |  |
| Subtotal |  |  |  | 85 | 23.94% |
| C | 00 | 10 | 10 | 9 |  |
|  | 01 | 00 | 00 | 7 |  |
|  | 00 | 11 | 11 | 13 |  |
|  | 10 | 00 | 00 | 11 |  |
|  | 11 | 10 | 10 | 13 |  |
|  | 10 | 11 | 11 | 10 |  |
|  | 00 | 01 | 01 | 5 |  |
|  | 11 | 00 | 00 | 6 |  |
|  | 01 | 11 | 11 | 7 |  |
|  | 11 | 01 | 01 | 5 |  |
|  | 01 | 10 | 10 | 1 |  |
|  | 10 | 01 | 01 | 1 |  |
| Subtotal |  |  |  | 88 | 24.79% |
| D | 11 | 11 | 10 | 16 |  |
|  | 11 | 11 | 01 | 4 |  |
|  | 01 | 01 | 00 | 2 |  |
|  | 11 | 11 | 00 | 4 |  |
|  | 10 | 10 | 00 | 2 |  |
|  | 11 | 01 | 00 | 5 |  |
|  | 01 | 11 | 00 | 5 |  |
|  | 10 | 01 | 00 | 3 |  |
|  | 11 | 10 | 00 | 1 |  |
|  | 10 | 11 | 00 | 3 |  |
|  | 01 | 10 | 00 | 4 |  |
| Subtotal |  |  |  | 49 | 13.80% |
| E | 00 | 00 | 10 | 8 |  |
|  | 00 | 00 | 01 | 3 |  |
|  | 00 | 00 | 11 | 4 |  |
|  | 01 | 00 | 11 | 3 |  |
|  | 01 | 01 | 11 | 3 |  |
|  | 01 | 10 | 11 | 1 |  |
|  | 10 | 00 | 11 | 2 |  |
|  | 10 | 01 | 11 | 4 |  |
|  | 10 | 10 | 11 | 1 |  |
|  | 00 | 10 | 11 | 4 |  |
|  | 00 | 01 | 11 | 1 |  |
| Subtotal |  |  |  | 34 | 9.58% |
| F | 11 | 00 | 10 | 11 |  |
|  | 11 | 00 | 01 | 5 |  |
|  | 00 | 11 | 01 | 3 |  |
|  | 00 | 11 | 10 | 9 |  |
| Subtotal |  |  |  | 28 | 7.89% |
| G | 00 | 01 | 10 | 2 |  |
|  | 00 | 10 | 01 | 1 |  |
|  | 10 | 11 | 01 | 5 |  |
|  | 10 | 00 | 01 | 4 |  |
|  | 10 | 10 | 01 | 1 |  |
|  | 01 | 01 | 10 | 3 |  |
|  | 11 | 01 | 10 | 3 |  |
|  | 11 | 10 | 01 | 6 |  |
| Subtotal |  |  |  | 25 | 7.04% |
| Total |  |  |  | 355 | 100% |

RCC, red crucian carp; CC, common carp; AT, allotatraploid.

The value 1 represents the presence of a band, while 0 represents the absence of a band. So, an amplification pattern of type 11 corresponds to samples displaying bands in both the MspI and HpaII lanes. Amplification patterns of the type 10 correspond to samples showing an amplified band after restriction with MspI but not after restriction with HpaII. Pattern 01 corresponds to samples displaying an amplified band after restriction with HpaII but not after restriction with MspI. Pattern 00 indicates no band amplified after restriction with either isoschizomer.
